# Suppression of Microgliosis With the Colony-Stimulating Factor 1 Receptor Inhibitor PLX3397 Does Not Attenuate Memory Defects During Epileptogenesis in the Rat

**DOI:** 10.3389/fneur.2021.651096

**Published:** 2021-06-03

**Authors:** Season K. Wyatt-Johnson, Alexandra L. Sommer, Kevin Y. Shim, Amy L. Brewster

**Affiliations:** ^1^Department of Psychological Sciences, Purdue University, West Lafayette, IN, United States; ^2^Weldon School of Biomedical Engineering, Purdue University, West Lafayette, IN, United States

**Keywords:** microglia, pilocarpine, epileptogenesis, hippocampus, memory, astrocytes, status epilepticus, complement-immunological terms

## Abstract

Events of status epilepticus (SE) trigger the development of temporal lobe epilepsy (TLE), a type of focal epilepsy that is commonly drug-resistant and is highly comorbid with cognitive deficits. While SE-induced hippocampal injury, accompanied by gliosis and neuronal loss, typically disrupts cognitive functions resulting in memory defects, it is not definitively known how. Our previous studies revealed extensive hippocampal microgliosis that peaked between 2 and 3 weeks after SE and paralleled the development of cognitive impairments, suggesting a role for reactive microglia in this pathophysiology. Microglial survival and proliferation are regulated by the colony-stimulating factor 1 receptor (CSF1R). The CSF1R inhibitor PLX3397 has been shown to reduce/deplete microglial populations and improve cognitive performance in models of neurodegenerative disorders. Therefore, we hypothesized that suppression of microgliosis with PLX3397 during epileptogenesis may attenuate the hippocampal-dependent spatial learning and memory deficits in the rat pilocarpine model of SE and acquired TLE. Different groups of control and SE rats were fed standard chow (SC) or chow with PLX3397 starting immediately after SE and for 3 weeks. Novel object recognition (NOR) and Barnes maze (BM) were performed to determine memory function between 2 and 3 weeks after SE. Then microglial populations were assessed using immunohistochemistry. Control rats fed with either SC or PLX3397 performed similarly in both NOR and BM tests, differentiating novel vs. familiar objects in NOR, and rapidly learning the location of the hidden platform in BM. In contrast, both SE groups (SC and PLX3397) showed significant deficits in both NOR and BM tests compared to controls. Both PLX3397-treated control and SE groups had significantly decreased numbers of microglia in the hippocampus (60%) compared to those in SC. In parallel, we found that PLX3397 treatment also reduced SE-induced hippocampal astrogliosis. Thus, despite drastic reductions in microglial cells, memory was unaffected in the PLX3397-treated groups compared to those in SC, suggesting that remaining microglia may be sufficient to help maintain hippocampal functions. In sum, PLX3397 did not improve or worsen the memory deficits in rats that sustained pilocarpine-induced SE. Further research is required to determine whether microglia play a role in cognitive decline during epileptogenesis.

## Introduction

Temporal lobe epilepsy (TLE) is a type of focal epilepsy that is commonly drug-resistant (1) and is highly comorbid with cognitive deficits (2). Cognitive comorbidities, including deficits in memory, attention, and executive function have been reported in 40–95% of patients with TLE (2). Unfortunately, currently available anti-seizure mediations do not address cognitive defects and in some cases can aggravate intellectual decline in individuals with epilepsy (3), supporting the need for novel therapies. In TLE, the hippocampus is often damaged as evidenced by the presence of extensive gliosis and severe neuronal loss, also known as hippocampal sclerosis. These disruptions to the hippocampal circuitry interfere with essential functions such as processing and consolidation of short- and long-term memories thereby resulting in learning and memory dysfunctions (2). Extensive research in animal models of acquired TLE, typically induced with a single episode of status epilepticus (SE), a long-lasting seizure (>1 h), support that SE-induced hippocampal injury in an otherwise healthy system contributes to epileptogenesis, as well as memory decline (4); though the mechanisms underlying memory loss after SE and TLE are still not fully understood. Our previous studies point to microglial cells, the resident immune cells of the brain, as potential mediators of learning and memory defects during SE-induced epileptogenesis (5–9).

Microglial proliferation and accumulation, microgliosis, in the hippocampus have been widely reported in human and experimental models of TLE, among other epilepsies (9, 10). Previously, we found a robust increase in microgliosis within the hippocampal CA1 region that peaked at 2 weeks post-SE (7, 11) and correlated with the development of hippocampal-dependent spatial learning and memory impairments (5, 6, 8). These microglia were characterized by bushy/amoeboid reactive morphologies (11) and activation of the mechanistic target of rapamycin (mTOR) signaling cascade (8). Importantly, we found that inhibition of mTOR signaling with the drug rapamycin during SE-induced epileptogenesis suppressed microgliosis and attenuated the associated memory loss (8). While this evidence suggests that reactive microglial cells may be active participants underlying cognitive dysfunctions in SE and TLE, mTOR signaling cascade is expressed in neurons and astrocytes (12), thereby indicating that these other cell types may also play a role in this pathophysiology.

Therefore, to specifically study the role of SE-induced microgliosis on cognitive decline we focused on the colony-stimulating factor 1 receptor (CSF1R) because CSF1R was identified by the computational casual reasoning analytical framework for target discovery as a potential anti-epileptic target (13). CSF1R is part of a family of receptors that are responsible for regulating microglial proliferation, survival, motility, and adhesion (14–16). As opposed to mTOR signaling, which is highly conserved across species and is ubiquitously found in numerous cell types (12), CSF1R is mainly expressed in microglia (14–16). In fact, mutations in this CSF1R receptor are associated with the loss of microglia and development of epilepsy in humans (17). In addition, inhibition of CSF1R with Plexxikon (PLX) 3397, or its analogs, have been shown to reduce/deplete the population of microglial cells as well as to improve behavioral cognitive performance in pre-clinical models of neurodegenerative disorders (16, 18–23). Therefore, in this study, we tested the hypothesis that suppression of microgliosis through inhibition of CSF1R signaling with PLX3397 during epileptogenesis may attenuate the hippocampal-dependent spatial learning and memory deficits in the rat pilocarpine model of SE and acquired TLE.

## Materials and Methods

### Animals

Male Sprague Dawley rats (150–200 grams) (Envigo) were housed (one animal per cage) at the Psychological Sciences Building at ambient temperature (22°C) with diurnal cycles of a 12-h light and 12-h dark (8:00–20:00), with access to unlimited food and water. Rats were weighed and handled daily. Animal procedures followed institutional and NIH guidelines and were approved by the Purdue Institutional Animal Care and Use Committee (Protocol #1309000927).

### Induction of SE

Pilocarpine was used to induce SE as previously described Schartz et al. (6), Schartz et al. (7), and Brewster et al. (8). Briefly, all rats were given an intraperitoneal (i.p.) injection of scopolamine methylbromide (1 mg/kg) 30 min (min) prior pilocarpine hydrochloride (280–300 mg/kg; i.p.) (*n* = 46) (SE rats) or a similar volume of saline (sham-treated controls, *n* = 26). The Racine scale (24) was used to score seizure stages from which stage 6 (rearing and falling) indicated the start of SE (*n* = 28). Following 1 h of SE, all rats were given diazepam (10 mg/kg; i.p.) and kept on heating pads. Chow was supplemented with chocolate Ensure and fruit loops for 1–3 days post-SE. Rats that did not reach SE were removed from the study (non-SE, *n* = 16). Two rats died during SE.

### PLX3397 Treatment

PLX3397 was mixed in rodent standard chow (SC) (455 mg PLX3397/kg of diet) (Envigo, Madison, WI) (National Research Council (US) Subcommittee on Laboratory Animal Nutrition 1995) to achieve a dose of 50 mg/kg body weight per day, which was found to be successful in reducing microglia in rats (25). A total of 27 rats received the PLX diet (Control, *n* = 13; SE, *n* = 14). Regular chow (SC) was given to 27 rats (Control, *n* = 13; SE, *n* = 14). After microglial counts were performed, animals on PLX diet that did not show reduced microgliosis compared to their respective control SC groups were deemed non-responders. Five rats from the SE+PLX group did not show decreases in the numbers of microglia and were classified as non-responders (SE+PLX Non-responder; Supplementary Figure 1). Therefore, these animals along with their tissues were not included in the behavioral, histological, or biochemical analyses. All control rats in the PLX diet showed reduced microglial populations relative to those in the C+SC group.

### Novel Object Recognition (NOR)

The NOR test was performed 2 weeks after SE following as previously described (5, 6). Rats were first acclimated in an adjacent dark room for 30 min before placed in the NOR test chamber (11.5 × 5.75 × 6 inches) for habituation (20 min). Twenty-four hours after habituation, NOR trials 1 and 2 were performed under red light conditions. In trial 1, rats were placed for 5 min in the testing chamber containing 2 identical objects. Two hours later, in trial 2, rats were placed for 5 min in the same chamber containing a familiar object from trial 1 and a novel object. The position of the novel and familiar objects in trial 2 was alternated to counterbalance potential side preferences. The Any-maze video-tracking system V4.99 (Wood Dale, IL) was used to record the NOR trials. The objects' exploration was measured when the rats' nose was within 1 cm from the objects. Time spent exploring the objects was determined by investigators blinded to experimental groups. The recognition index (RI) determined the difference in exploration time between the same object in trial 2 (familiar) and trial 1 relative to the total exploration time during both trials [(Trial1-Trial2)/Trial1+Trial2)]. NOR testing was completed in 4 cohorts (*N* = 10/group; sample size was determined a priori by G^*^Power, on a power of 80% and rats were selected randomly), three rats in the SE+PLX group were removed for non-response to PLX treatment following microglial counts, and two rats in SE+SC group were removed due to outliers in NOR index test (C+SC, *n* = 10; C+PLX, *n* = 10; SE+SC, *n* = 8; SE+PLX, *n* = 7).

### Barnes Maze (BM)

BM was performed after completion of the NOR test (16–20 days after SE), following previously described protocols (5, 6). Briefly, on BM day 1 (habituation), rats were trained to find and enter the escape box as follows: (1) rats were placed directly into the escape box; (2) rats were placed next to the escape box and gently nudged into the box; (3) rats were placed in the center of the platform and guided to the escape box through an open tunnel. On BM training days 1–4, rats were tested in 4 trials/day (15 min between trials) to find the escape box within 3 min or were gently guided into the box after the 3 min expired. On BM day 5 the escape box was removed, the holes were covered, and the rats were tested for their ability to find the location of the covered target hole within 90 s (probe trial). Any-maze video tracking system V4.99 (Wood Dale, IL) was used to record and measure the time to find the escape box during days 1–4 (escape latency), and time spent over the target covered hole during the probe trial. BM testing was completed in 4 cohorts (*N* = 10/group; sample size was determined a priori by G^*^Power, on a power of 80% and rats were selected randomly), three rats in the SE+PLX group were removed for non-response to PLX treatment following microglial counts (C+SC, *n* = 10; C+PLX, *n* = 10; SE+SC, *n* = 10; SE+PLX, *n* = 7).

### Perfusion

Rats were deeply anesthetized with a lethal dose of Beuthanasia (200 mg/kg) and perfused with ice-cold 1X phosphate-buffered saline (PBS; 137 mM NaCl, 2.7 mM KCl, 4.3 mM Na2HPO4, 1.47 mM KH2PO4, pH 7.4). The brain was removed and hemispheres separated. One hemisphere was post-fixed with 4% paraformaldehyde (PFA) (Thermo Fisher Scientific, Rockville, IL) for immunostainings. The hippocampus was dissected from the other hemisphere, and frozen at −80°C for protein extraction and immunoblotting.

### Immunohistochemistry (IHC)

Brain tissues fixed in PFA for 48 hours at 4°C were cryoprotected in 30% sucrose (Thermo Fisher Scientific) (diluted in 1X PBS), frozen in dry ice, and stored at −80°C until used. Coronal sections (20 μm) between the bregma coordinates: −3.00 mm to −5.28 mm were obtained using a Leica CM1860 cryostat and stored in 1XPBS + 0.1% sodium azide at 4°C until used. IHC was done in free-floating sections exactly as previously described (7, 11). Primary antibodies: anti-rabbit IBA1; Secondary antibodies: biotinylated goat anti-rabbit (1:1K; BA-1000, Vector Laboratories, Burlingame, CA) for 1 h at RT. Immunosignal was developed using the ABC Avidin/Biotin complex solution and DAB Peroxidase (HRP) Substrate Kit, 3,3′-diaminobenzidine (SK-4100, Vector Laboratories), and visualized using a Leica DM500 microscope. Images were captured with a high-resolution digital camera (Leica MC120 HD) with 4X and 40X objectives using the LAS4.4 software. Three to six brain sections per rat were analyzed.

### Microglia Counts

Cells were counted, exactly as previously described Wyatt-Johnson et al. (11), from the hippocampal CA1 region, and all IBA1-positive cells within the entire 40X image were counted. For this experiment, IBA1-positive cells included both microglia and macrophages referred to throughout this paper as microglia (26). Exclusion guidelines were set: (1) 25% or more of the IBA1-positive cells were located outside the boundaries of the counting region; (2) region of the tissue was broken; (3) stain was too light to visualize. Three distinct, non-overlapping images in CA1 region, 3–6 sections per rat were analyzed. Cell counts were performed, three rats in the SE+PLX group were removed for non-response to PLX treatment (C+SC, *n* = 13; C+PLX, *n* = 13; SE+SC, *n* = 14; SE+PLX, *n* = 9).

### Western Blot (WB)

Hippocampi were homogenized in ice-cold 1XPBS and processed for immunoblotting as previously described Schartz et al. (6) and Brewster et al. (8). The Bradford Protein Assay (Bio-Rad, Hercules, CA) was used to determine protein concentration. Samples were diluted with Laemmli buffer (0.25 M Tris, pH 6.8, 6% sodium dodecyl sulfate (SDS), 40% sucrose, 0.04% Bromophenol Blue, 200 mM Dithiothreitol), separated via SDS-PAGE in Tris-glycine gels (7, 10, or 15%), and transferred to polyvinylidene fluoride membranes (Cat# 88518, GE Healthcare, Chicago, IL). Then, membranes were blocked with 5% non-fat milk diluted in 1XPBS+ 0.1% Triton at RT for 1 h on a rocking platform. Membranes were incubated with primary antibodies anti-rabbit CX3CR1 (1:1K; ab8021; Abcam, Cambridge, United Kingdom), anti-mouse GFAP (1:50K; #3670; Cell Signaling), anti-goal C3 (1:500; 855730; MP Biomedical, Solon, OH), or anti-mouse Beta-Actin (1:5K; #3700S; Cell Signaling) at 4°C overnight. Following multiple washes in 1XPBS with 0.1% Tween, membranes were incubated with HRP-linked secondary antibodies anti-rabbit (1:2K; ab205718; Abcam), anti-mouse (1:2K; ab205719; Abcam), or anti-goat (1:5K; AP180P; Millipore, Burlington, MA). Immunoreactive bands were visualized using enhanced chemiluminescence prime western blotting detection reagent (GE Healthcare), captured on Double Emulsion Blue Autoradiography Film (BX57, MIDSCI, St. Louis, MO). Membranes were stripped from primary antibodies using stripping buffer (25 mM glycine, pH 2.0, 10% SDS) for 2 h at RT then washed in 1XPBS with 0.1% Tween and re-blotted with primary antibody as described above.

### Densitometry Analysis for Western Blot

The relative pixel density of each of the immunoreactive bands was measured with the Image J software V1.49 (NIH) (8). Background signal was recorded and subtracted from the immunoreactive bands. Proteins of interest were normalized to the loading control in the same lane/sample. Only the animals where PLX treatment was deemed successful, were included in the analysis (C+PLX, n=13; SE+PLX, *n* = 9), and any samples that had artifacts such as bubbles or smudges in the immunoblots were not included in the final analysis (CX3CR1 & GFAP: C+SC, *n* = 13; C+PLX, *n* = 11; SE+SC, *n* = 14; SE+PLX, *n* = 9; C3 & iC3b: C+SC, *n* = 9; C+PLX, *n* = 7; SE+SC, *n* = 8; SE+PLX, *n* = 8).

### Statistical Analysis

G^*^Power was used for a priori analysis to determine sample sizes with previously collected data based on a power of 80%. Student's *t*-test was used to analyze comparisons between two groups. Two-way ANOVA with Tukey's multiple comparisons was used to compare 4 groups (C+SC, C+PLX, SE+SC, and SE+PLX). Three-way mixed effects model (days/time vs. condition vs. treatment) with Tukey's multiple comparisons test was used for bodyweight comparisons. Three-way ANOVA (condition vs. treatment vs. training block) with Tukey's multiple comparison test was used to analyze BM. Kolmogorov-Smirnov test was used to analyze the non-normally distributed Racine scale data. The Kruskal-Willis test with Dunn's multiple comparisons was used to analyze the non-normally distributed microglial morphological data and the Racine scale data from the SE+PLX Non-responders. A one-way ANOVA with Dunnett's multiple comparison test was used to analyze the differences between seizure threshold and microglial responses between the SE+PLX Non-responders, SE+SC and SE+PLX responders groups (Supplementary Figure 1). Two-way ANOVA with Dunnett's multiple comparison test was used to analyze Non-responders weight changes compared to the SE+SC and SE+PLX groups (Supplementary Figure 1). Outliers were determined with ROUT with the maximum desired false recovery rate set to 1%, if an outlier was removed in one test, it was removed in all other dependent tests, this only occurred with the NOR analysis. Statistical significance was set at α < 0.05. Data values were reported as mean ± standard error of the mean (SEM). GraphPad Prism 6 software was used for statistical analyses. Figures were generated using Adobe Photoshop (CS6) and Biorender.com.

## Results

**Figure 1:**
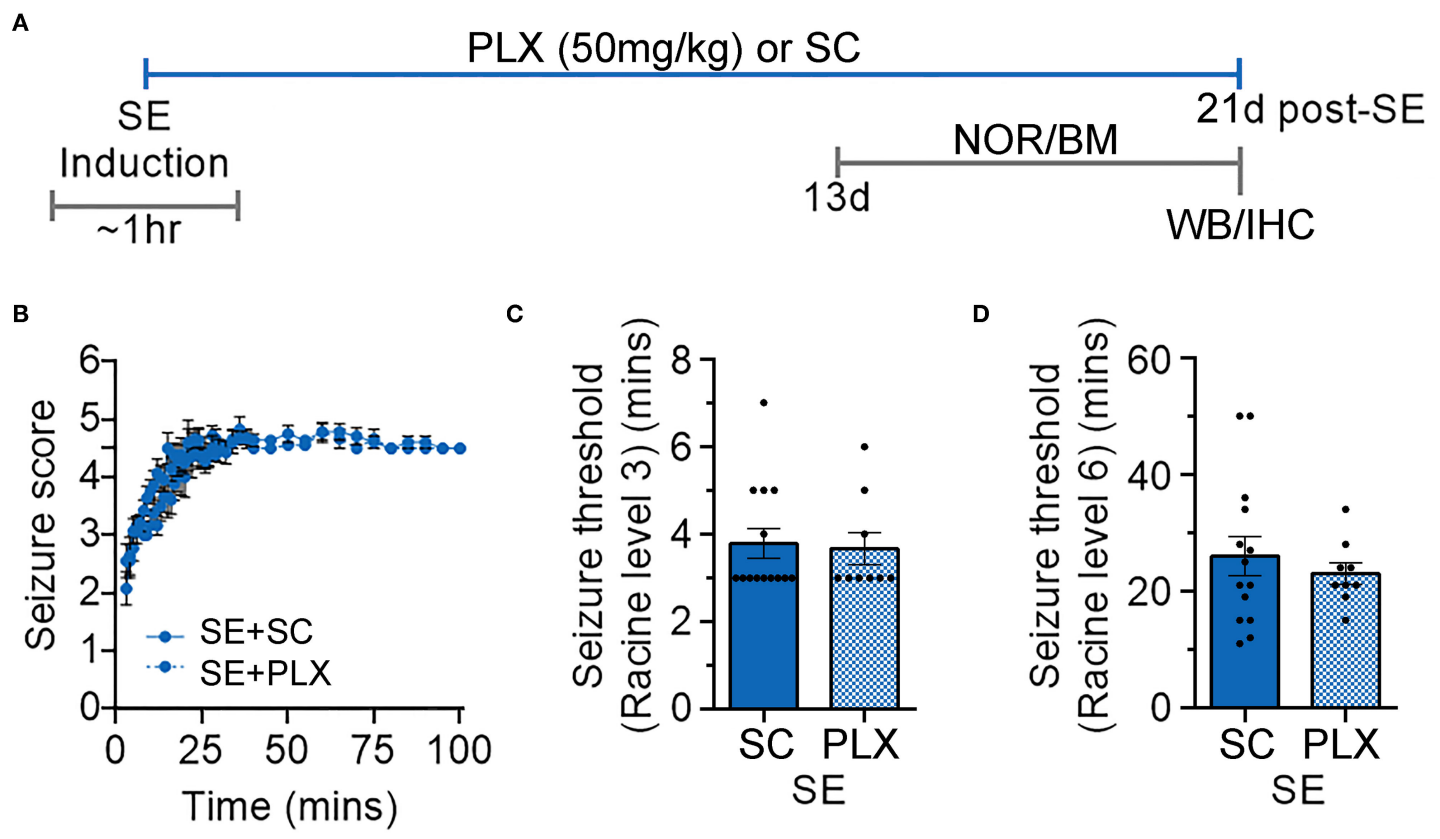
Timeline of induction of status epilepticus (SE) and experimental design. **(A)** Diagram with the timeline of the experimental design with treatments with PLX3397 in chow (PLX; 50 mg/kg per day) or standard chow (SC) alone. Different groups of rats were given PLX or SC starting immediately after SE induction (day 0) to up to 20 days thereafter. Novel object recognition (NOR) and Barnes maze (BM) were performed between days 13 and 20. Brain tissues were then collected for histological and biochemical analyses on experimental day 21. **(B)** Behavioral seizures were monitored for 100 min after SE induction and scored according to the Racine scale (1: rigid posture, mouth moving; 2: tail clonus; 3: partial body clonus, head bobbing; 4: rearing; 4.5: severe whole body clonic seizures while retaining posture; 5: rearing and falling; 6: tonic-clonic seizure with jumping or loss of posture). **(C)** Time to first seizure (level 3). **(D)** Time to SE (level 6). Data analyzed by Kolmogorov-Smirnov test **(B)** and student's *t*-test **(C,D)**. Data are shown as mean ± SEM, SE+SC, *n* = 14; SE+PLX, *n* = 9.

SE and control rats were randomly assigned to different groups fed with either a rodent standard chow (SC) or chow with PLX3397 (50 mg/kg) for 20 days (C+SC, C+PLX, SE+SC, and SE+PLX). Rats were then exposed to a series of behavioral tests including NOR and BM, followed by brain tissue collection for histological or biochemical processing (Figure 1A). During SE inductions the seizures, which were scored according to the Racine scale (24), were not different between rats assigned to either diet [Kolmogorov-Smirnov test, *D* = 01556, *p* = 0.6476] (Figure 1B). No differences were found in the time to reach level 3 (first seizure) [student's *t*-test, *t*_(21)_ = 0.2318, *p* = 0.8190; Figure 1C], or in the time to level 6 (SE) [student's *t*-test, *t*_(21)_ = 0.6675, *p* = 0.5117; Figure 1D]. These data indicate that rats assigned to the CS or PLX treatments reached similar SE severities.

### PLX Does Not Attenuate SE-Induced Weight Loss

**Figure 2:**
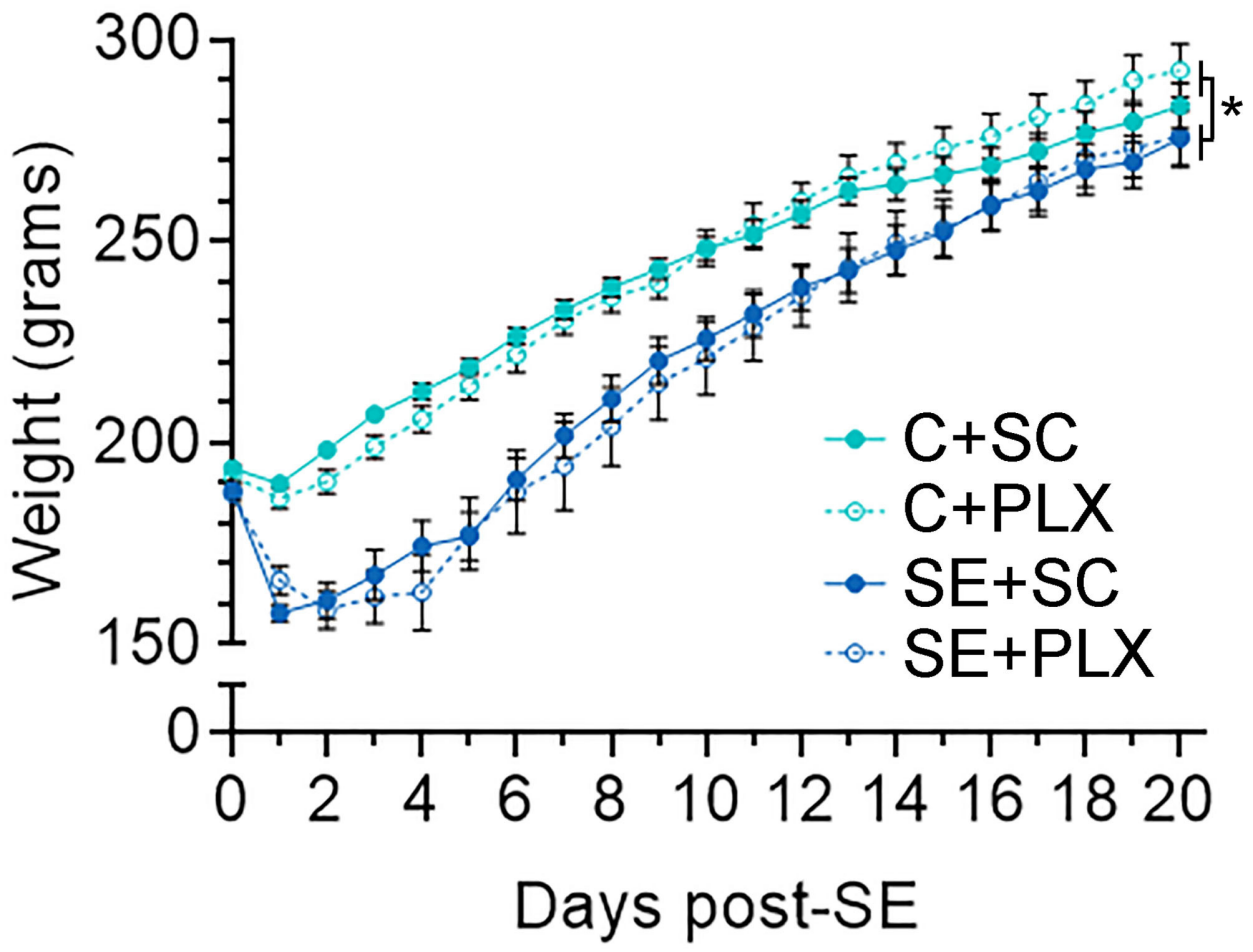
PLX3397 (PLX) treatment does not alter body weight gain in control or SE rats. Graph shows the daily boy weight of rats (days 0–21) from the control and SE groups fed with either chow with PLX or standard chow alone (SC). Data are shown as mean ± SEM. *p < 0.05 by a three-way mixed effects model with Tukey's multiple comparisons test. C+SC, *n* = 13; C+PLX, *n* = 13; SE+SC, *n* = 14; SE+PLX, *n* = 9.

Immunomodulating drugs have been shown to promote weight recovery after SE (5, 27, 28). Therefore, to determine if PLX treatment has an effect on bodyweight, we monitored the rats' weight daily during this study (Figure 2). All rats had similar weights prior to pilocarpine or saline injections (day 0). One day after SE, all SE rats lost weight while control rats gained weight (SE+SC: −3.21 ± 3.88 g; SE+PLX: −7.33 ± 2.36 g; C+SC: +8.34 c± 0.75 g; C+PLX: 4.07 ± 0.99 g). Thereafter, all rats gained weight over time [Mixed-effects ANOVA, *F*_(20,900)_ = 911.1, *p* < 0.0001] [C+SC: 4.93 ± 0.46 g/day (Mean ± SEM); SE+SC: 6.20 ± 0.69 g/day; C+PLX: 5.19 ± 0.46 g/day; SE+PLX: 5.807 ± 1.04 g/day]. However, between 1 and 7 days after SE, the bodyweight of the SE+SC and SE+PLX rats remained significantly lower relative to the C+SC or C+PLX groups (*p* < 0.05). On day 21, the body weight was similar in all groups (*p* = 0.99). Taken together, these data suggest that SE resulted in a transient decrease in body weight that normalized by 3 weeks after SE, and that PLX had no effect on the weight gain of either control or SE rats.

### PLX Treatment Does Not Alter Recognition Memory in Rats

**Figure 3:**
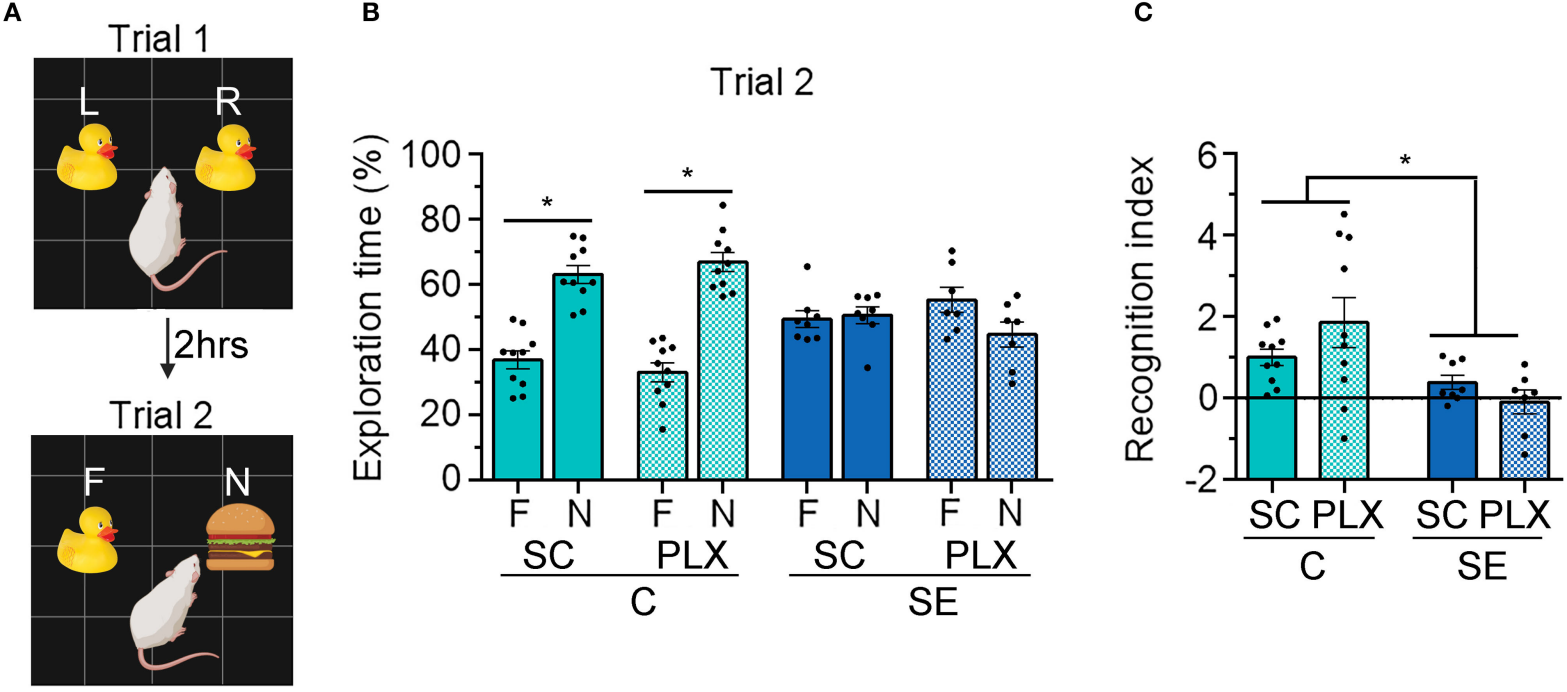
PLX3397 (PLX) treatment after status epilepticus (SE) does not attenuate recognition memory impairments assessed with the novel object recognition (NOR) test. **(A)** Representative illustration of the NOR test trials 1 and 2. **(B)** Percentage exploration time of the familiar (F) and the novel objects (N) in trial 2 is shown. **(C)** Recognition index of the familiar objects in trials 1 and 2 [(F1-F2)/(F1+F2)] is shown. Data are shown as mean ± SEM. *p < 0.05 by student's *t*-test **(B)** and two-way ANOVA with Tukey's multiple comparisons test **(C)**. SC, standard chow. C+SC, *n* = 10; C+PLX, *n* = 10; SE+SC, *n* = 8; SE+PLX, *n* = 7.

**Figure 4:**
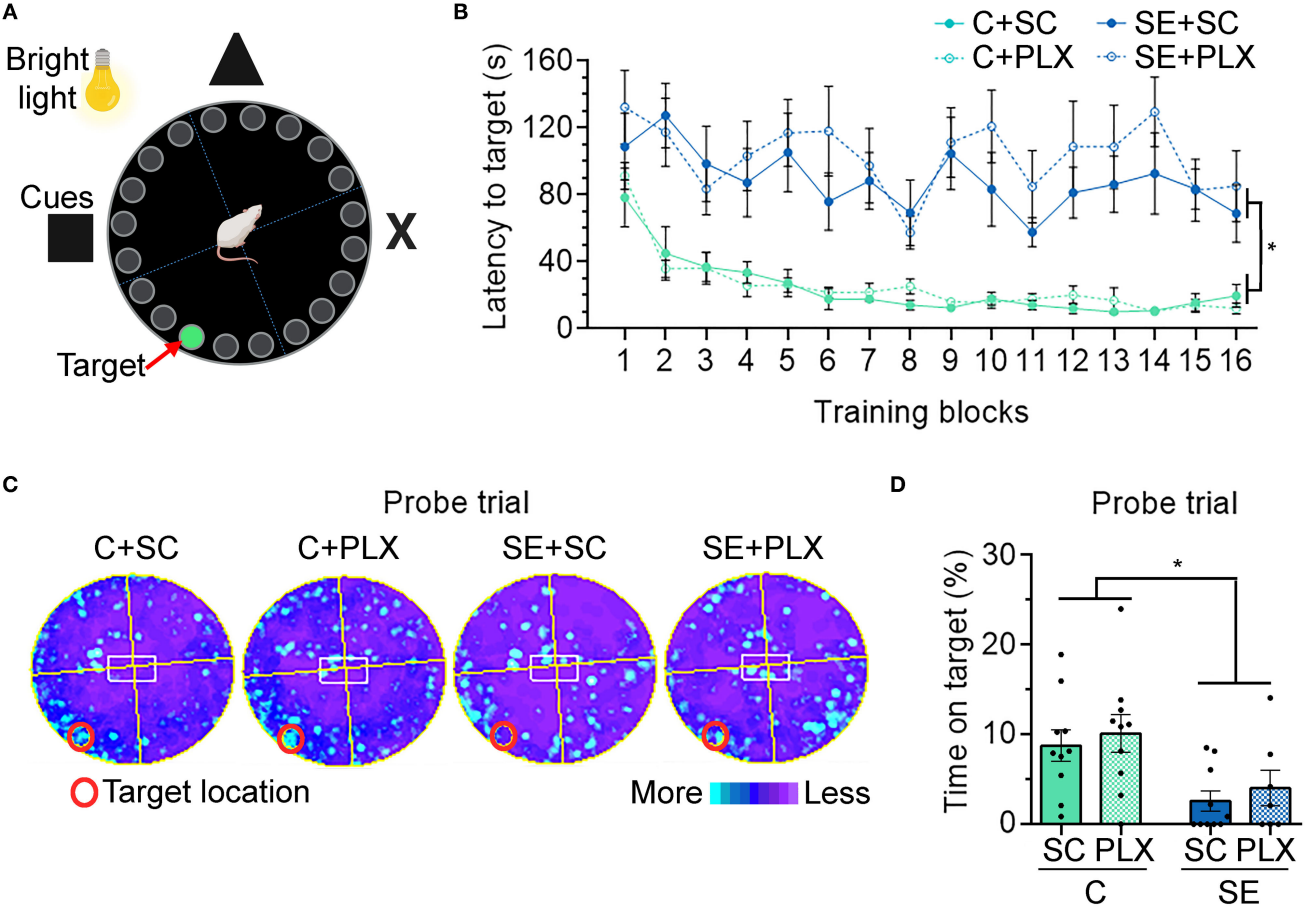
PLX3397 (PLX) treatment after status epilepticus (SE) does not attenuate hippocampal-dependent spatial learning and memory deficits assessed with the Barnes maze (BM). **(A)** Representative illustration of the BM. **(B)** Graph shows the latency to find the target hole on the BM platform during training days 1–4 (4 trials/day). **(C)** Heat maps are shown as the average time spent in each location during the probe trial test. Red circle indicates the target location on the platform (covered hole). (**D)** Percentage of time spent on the target during the probe trial. Data are shown as mean ± SEM. *p < 0.05 by three-way ANOVA with Tukey's multiple comparisons **(B)** and two-way ANOVA with Tukey's multiple comparisons **(D)**. SC, standard chow. C+SC, *n* = 10; C+PLX, *n* = 10; SE+SC, *n* = 10; SE+PLX, *n* = 7.

SE is often followed by memory impairments which parallel microgliosis in the hippocampus (5, 6, 8). Previously, we found that administration of the drug rapamycin during SE-induced epileptogenesis suppressed microgliosis and attenuated hippocampal-dependent learning and memory deficits (8). Thus, to further assess the role that microgliosis may play in the SE-induced cognitive decline we used NOR (Figure 3) and BM (Figure 4) to test recognition and spatial memory, respectively, in control and SE rats treated with SC or PLX. NOR was performed 2 weeks post-SE, when cognitive impairments are evident (5, 6, 8), and when spontaneous behavioral seizures occur with a frequency of ~1.5 seizures per 48 h (2–3 weeks after SE) (6). Following the familiarization trial 1, rats were exposed to a familiar and a novel object in trial 2 (Figure 3A). We found that rats in the C+SC or C+PLX groups spent significantly more time exploring the novel object compared to the familiar object [C+SC: *t*_(18)_ = 6.750, *p* < 0.0001; C+PLX: *t*_(18)_ = 8.180, *p* < 0.0001]. In contrast, rats in the SE+SC or SE+PLX groups spent similar amounts of time exploring both objects [SE+SC: *t*_(14)_ = 0.3224, *p* = 0.7519; SE+PLX: *t*_(12)_ = 1.934, *p* = 0.0770] (Figure 3B), suggesting that these SE rats did not remember the familiar object from trial 1. In addition, we determined the RI, which indicates the extent to which the familiar object was recognized in trial 2. The C+SC and C+PLX groups had significantly greater RI than the SE+SC or SE+PLX groups [Main effect, two-way ANOVA, *F*_(1,30)_ = 15.32, *p* = 0.0005] (Figure 3C), suggesting that the control animals treated with CS or PLX had greater memory of the familiar objects compared to both SE groups. Taken together these findings indicate that SE provokes memory defects and that PLX treatment had no effect on the recognition memory of either control or SE rats.

### PLX Treatment Does Not Alter Hippocampal-Dependent Learning and Memory in Rats

Following NOR, rats were tested for hippocampal-dependent spatial learning and memory using the BM (Figure 4). Rats were tested for their ability to find an escape hole (latency to target) in a circular platform by relying on spatial navigation cues (Figure 4A). During the first block, the latency to reach the target was similar in all groups (*p* > 0.9999) (Figure 4B). Thereafter, the latency to target decreased with each subsequent block [main effect blocks, three-way ANOVA, *F*_(15,528)_ = 4.485, *p* < 0.0001], suggesting general learning of the target location over time. However, rats from the C+SC and C+PLX groups showed a significant decrease in latency to target when compared to the SE+SC or SE+PLX groups [main effect condition, three-way ANOVA, *F*_(1,528)_ = 410.4, *p* < 0.0001], suggesting a SE-induced learning deficit that was not attenuated with PLX. On the probe trial to test memory retention (Figures 4C,D), the C+SC and C+PLX groups spent significantly more time over the covered target hole when compared to the SE+SC and SE+PLX groups [two-way ANOVA, *F*_(1,33)_ = 11.74, *p* = 0.002], suggesting a memory deficit in both SE groups that was not attenuated with PLX. Taken together, these findings confirm that SE-induces deficits in hippocampal-dependent spatial learning and memory that are evident during epileptogenesis, and that PLX treatment did not have an effect on cognitive functions in control or SE rats.

### PLX Treatment Attenuated SE-Induced Microgliosis in the Ca1 Hippocampus

**Figure 5:**
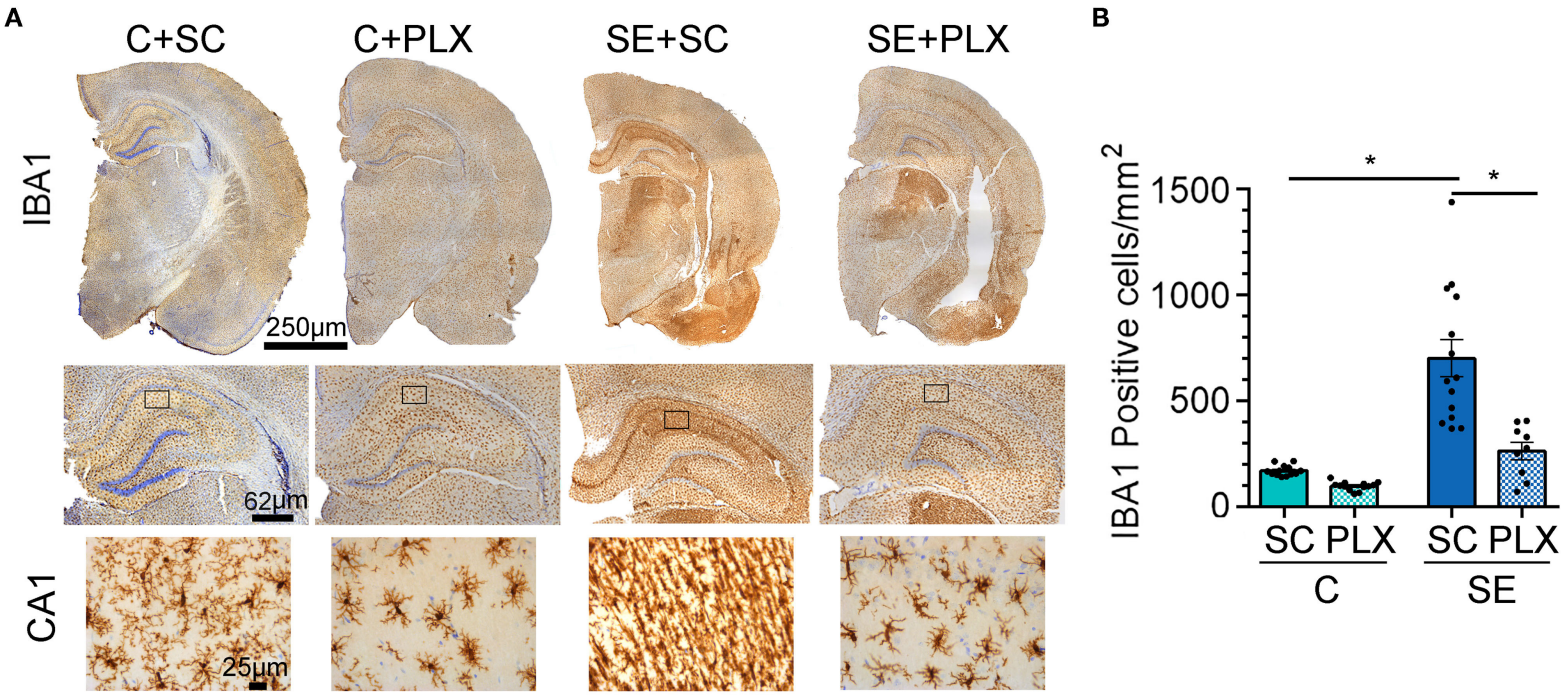
PLX3397 (PLX) treatment after status epilepticus (SE) reduces microgliosis in the hippocampus. **(A)** Representative IBA1 (brown) and Nissl-stained cellular nuclei (blue) are shown at low magnification for one hemisphere (4x) and higher magnetization for the hippocampus (20X) and associated CA1 stratum radiatum (sr) region (boxed- 40X). **(B)** Quantification of IBA1 positive cells per mm^2^ in CA1 sr. Data are shown as mean ± SEM. *p < 0.05 by two-way ANOVA with Tukey's multiple comparisons. SC, standard chow. C+SC, *n* = 13; C+PLX, *n* = 13; SE+SC, *n* = 14; SE+PLX, *n* = 9.

Microgliosis is a hallmark of epileptogenesis and has been thoroughly characterized in experimental models of SE (9). Previously, we found that microgliosis peaked in the hippocampal CA1 region between 2 and 3 weeks after SE (7, 11). Thus, following behavioral tests, we determined the extent to which PLX suppressed microgliosis in this brain region, as a representative area, by performing counts of IBA1-positive microglial cells (Figure 5). Consistent with our previous reports, significantly increased numbers of microglial cells were evident in the SE+SC group when compared to the C+SC group [*F*_(1,45)_ = 43.22, *p* < 0.0001]. Compared to these groups, the number of microglial cells was significantly reduced in both C+PLX and SE+PLX groups [interaction, two-way ANOVA *F*_(1,45)_ = 11.96, *p* = 0.001]. The number of microglial cells was reduced by 60% in the C+PLX group compared to the C+SC group [student's *t*-test, *t*_(24)_ = 8.148, *p* < 0.0001]. Similarly, the number of microglial cells was reduced by 60% in the CA1 region of the SE+PLX group relative to the SE+SC group [*t*_(21)_ = 3.858, *p* = 0.0009]. In addition, there was a sub-group of rats within the SE group that did not respond to the PLX treatment [one-way ANOVA *F*_(2,25)_ = 20.40, *p* < 0.001] (Supplementary Figure 1). The number of microglial cells was significantly higher in the SE+PLX Non-responder group compared to the SE+PLX responder group (by ~300%; *p* < 0.0001) or the SE+SC (by 60%; *p* = 0.0033; Supplementary Figure 1). Together these data indicate that a 3-week treatment with PLX mixed in chow was effective at reducing the SE-induced microglial proliferation in the CA1 hippocampal region.

**Figure 6:**
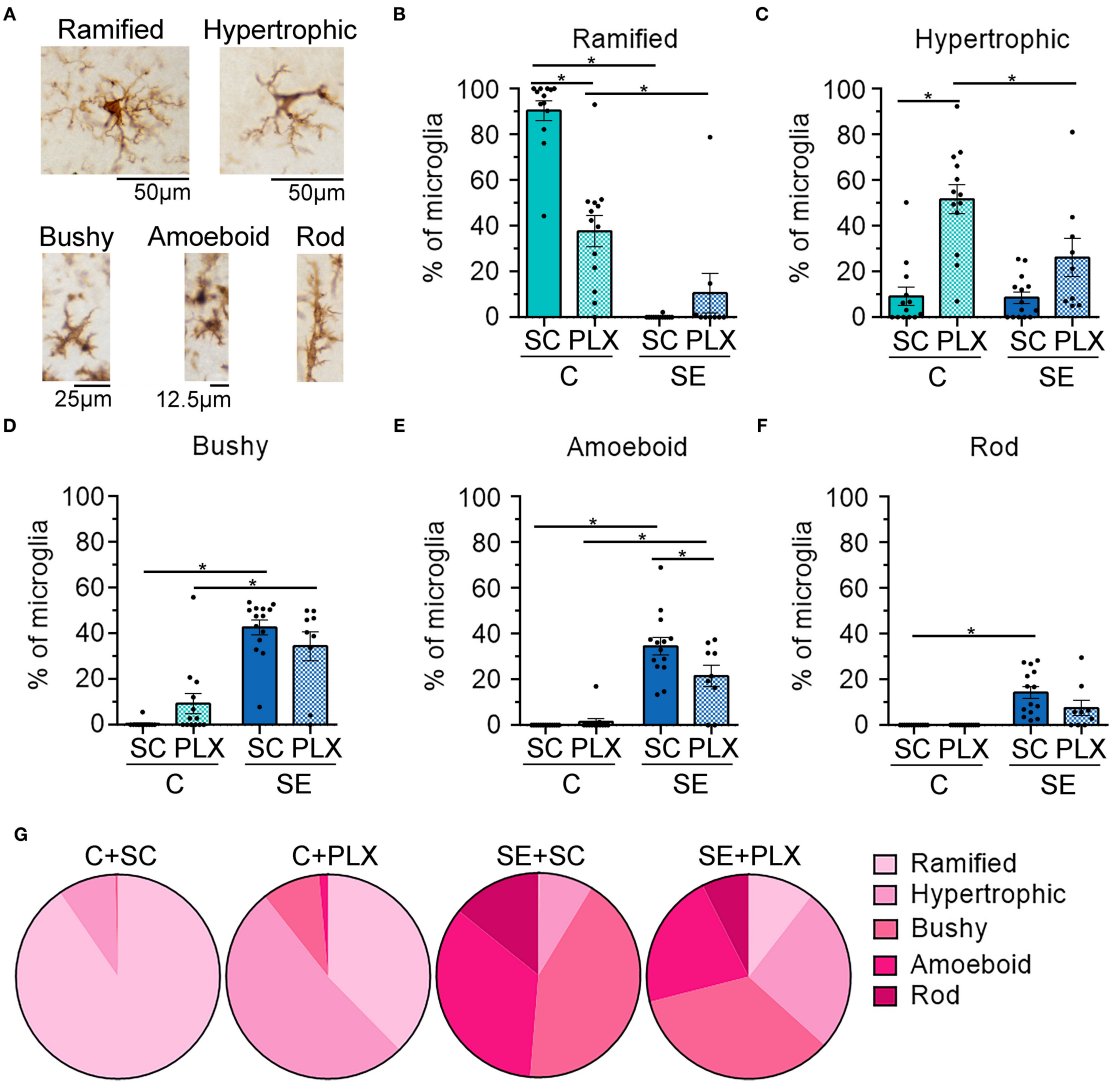
PLX3397 (PLX) treatment alters the morphology of microglia in control and SE rats. **(A)** Representative images of different microglial (brown) morphologies observed in the hippocampus. **(B–F)** Morphological breakdown of each group as ramified **(B)**, hypertrophic **(C)**, 3-bushy **(D)**, amoeboid **(E)**, and rod **(F)**. **(G)** Pie charts are shown the microglial morphologies in each treatment group. *p < 0.05 by Kruskal-Wallis test with Dunn's multiple comparisons. Data are shown as mean ± SEM. SC, standard chow. C+SC, *n* = 13; C+PLX, *n* = 13; SE+SC, *n* = 14; SE+PLX, *n* = 9.

Next, we determined the effects of PLX on the morphology of the remaining microglial cells because following SE hippocampal microglia display an array of different shapes that evolve during epileptogenesis (Figure 6) (11). We found that PLX treatment altered the morphology of remaining microglial cells within the CA1 hippocampi of rats from both C+PLX and SE+PLX groups. We organized microglia into five distinct shapes: ramified, hypertrophic, bushy, amoeboid, and rod morphologies, based on the diameter of the cell, and the length and thickness of the processes (Figure 6A) (11). In the C+SC group, there was a significant difference among these microglial morphologies [Kruskal-Wallis test*, H*_(5,65)_ = 51.07, *p* < 0.0001], with higher numbers of ramified microglia compared to the other four morphologies (Figures 6B–G). In contrast, in the C+PLX group, the shapes of the remaining microglia were mainly ramified and hypertrophic [Kruskal-Wallis test*, H*_(5,65)_ = 45.41, *p* < 0.0001], suggesting that PLX had an effect on the remaining microglia. Consistent with our previous findings, the SE+SC group had increased numbers of microglial cells with bushy and amoeboid shapes compared to ramified, hypertrophic, or rod [Kruskal-Wallis test*, H*_(5,65)_ = 53.34, *p* < 0.0001]. In the SE+PLX group, microglial morphologies were mainly hypertrophic, bushy, or amoeboid [Kruskal-Wallis test*, H*_(5,50)_ = 13.88, *p* = 0.008]. Overall, PLX had an effect on the morphology of remaining microglia in both control and SE groups, with a shift to a larger population of cells with hypertrophic shapes suggesting the possibility of a change in the functional status of the remaining microglial cells.

### PLX Attenuated SE-Induced Astrogliosis in the Hippocampus

**Figure 7:**
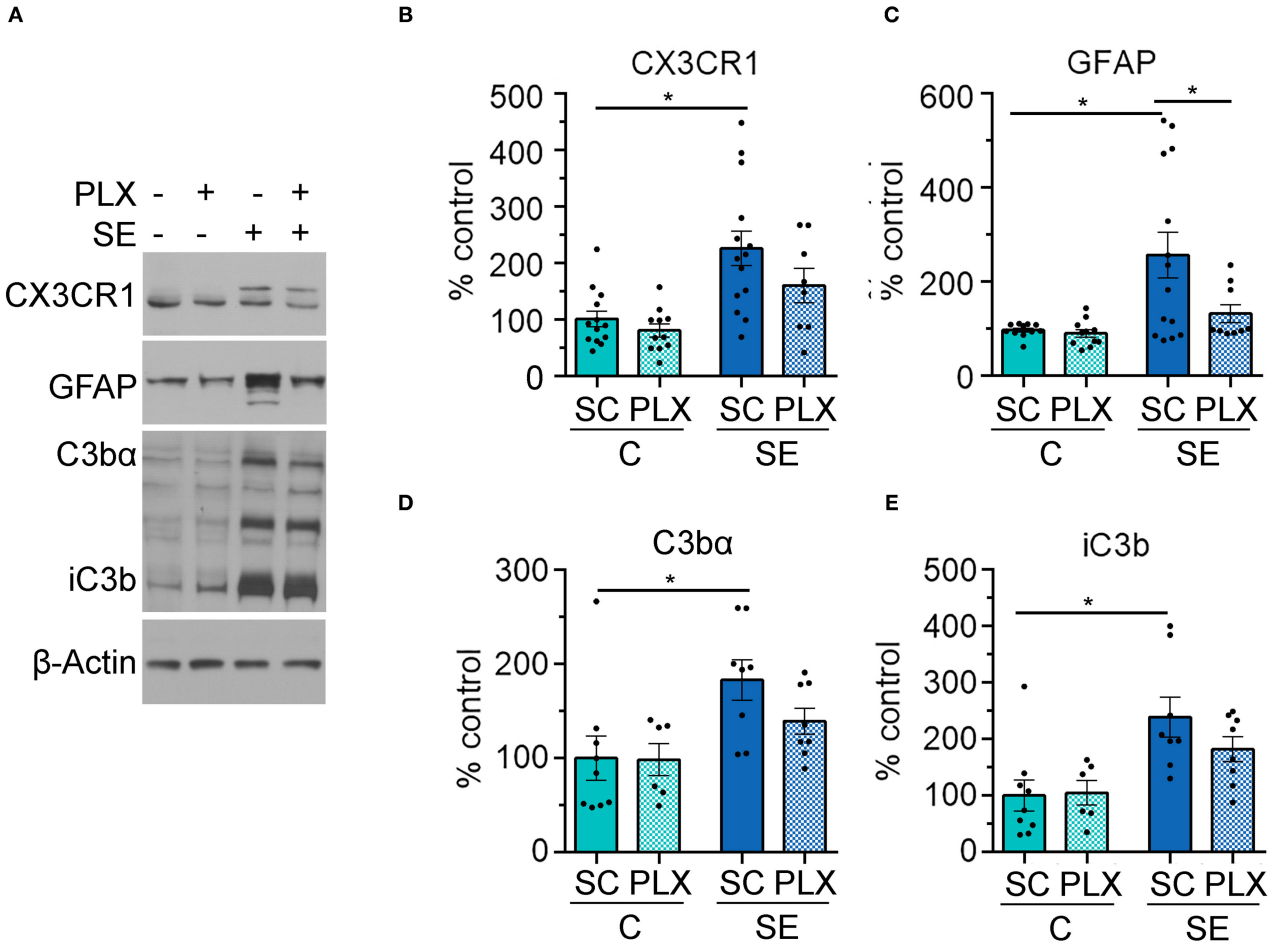
PLX3397 (PLX) treatment after status epilepticus (SE) reduces astrogliosis in the hippocampus. **(A)** Representative immunoblots with antibodies against CX3CR1, GFAP, C3bα, and iC3b and corresponding β-Actin (loading control). **(B–E)** Quantitative analysis of the mean pixel intensity shown as % control for CX3CR1 **(B)**, GFAP **(C)**, C3bα **(D)**, and iC3b **(E)**. *p < 0.05 by two-way ANOVA with Tukey's multiple comparisons. Data are shown as mean ± SEM. SC, standard chow. C+SC, *n* = 9–13; C+PLX, *n* = 7–11; SE+SC, *n* = 8–14; SE+PLX, *n* = 8–9.

To further assess the effects of PLX treatment on microglia we determined levels of the CX3C chemokine receptor 1 (CX3CR1) which is expressed in microglia (29, 30). In parallel, we determined levels of the complement protein C3 and its biologically active fractions because these are produced/released by astrocytes in response to microglial signals (31, 32) and are increased by SE (5, 6, 32). We found significantly increased levels of CX3CR1 protein in hippocampi of SE+SC rats when compared to the C+SC group [Figure 7B, main effect condition, two-way ANOVA, *F*_(1,42)_ = 17.97, *p* = 0.0001] (C+SC vs. SE+SC, *p* = 0.001). However, there was no significant difference in the protein levels of CX3CR1 between the SE+SC and SE+PLX groups (*p* = 0.26), or between C+SC and C+PLX groups (*p* = 0.92). While PLX reduced the number of microglial cells in CA1 (Figure 5), it did not alter the levels of this fractalkine receptor suggesting the possibility that there may be a compensatory increase in CX3CR1 expression in the remaining microglial cells in the SE+PLX group. Next, we determined the protein levels of the glial fibrillary acidic protein (GFAP) found in astrocytes, as well as the protein levels of the complement C3 fractions C3bα and iC3b. We found a significant increase in the levels of GFAP, C3bα, and iC3b in hippocampi of SE+SC rats when compared to the C+SC group (GFAP: *p* = 0.003; C3bα: *p* = 0.02; iC3b: *p* = 0.006). While PLX treatment significantly decreased the GFAP protein levels in the SE+PLX group when compared to the SE+SC group (*p* = 0.04), it did not alter GFAP levels in the C+PLX group relative to the C+SC group (*p* = 0.99) (Figure 7C). PLX treatment did not alter the protein levels of C3bα (Figure 7D) or iC3b (Figure 7E) in either the C+PLX or SE+PLX groups when compared to C+SC and SE+SC, respectively (C3: SE+PLX vs. C+SC, *p* = 0.48; SE+PLX vs. SE+SC, *p* = 0.41; iC3b: SE+PLX vs. C+SC, *p* = 0.99; SE+PLX vs. SE+SC, *p* = 0.47). These data suggest that in addition to reducing SE-induced microgliosis PLX treatment reduced astrogliosis in hippocampi of SE rats.

## Discussion

To understand the contribution of microglial cells to the cognitive dysfunction associated with epileptogenesis we used a dietary treatment with the drug PLX3397 to decrease the population of microglial cells during this critical period. The main findings of this study are that the SE-triggered cognitive defects were not resolved with PLX3397 treatment (Figures 3, 4), even though PLX3397 significantly reduced both SE-induced microgliosis (Figure 5) and astrogliosis in the hippocampus (Figure 7C).

The use of new pharmacological tools such as PLX3397 and its analogs, that specifically suppress CSF1R signaling to interrupt microglial survival/proliferation and reduce their population, implicate these cells as essential players underlying memory defects in preclinical models of neurodegeneration (19–23). For instance, reduction in the population of microglial cells with a PLX3397 or PLX5622 diet restored contextual memory in mouse models of Alzheimer's disease (AD) (19, 20), improved spatial memory in aged mice (22), and attenuated radiation-induced cognitive deficits in mice (21). Based on this evidence we expected that reductions of SE-induced microgliosis with PLX3397 during epileptogenesis would improve cognitive functions. However, despite significant reductions in the numbers of microglia triggered by the PLX3397 diet in both control and SE rats (Figure 5), cognitive behaviors remained similar to the rats on regular chow (Figures 3, 4). This suggests the possibility that the remaining microglia are sufficient to help maintain circuit functions, or that the roles of microglia vary in different disorders, such as in epilepsy which is characterized by neuronal hyperactivity and seizures.

The presence of reactive microglia with an array of morphologies and cytokine profiles with diverse spatiotemporal profiles have been widely described in response to SE as well as in chronic epilepsy (9, 10, 33). However, whether this microgliosis is beneficial or detrimental during epileptogenesis or in established epilepsy is still unresolved. In some models of acquired epilepsies, as well as in models of brain injury by trauma or stroke, drastic reductions in microglial populations with CSF1R inhibitors were shown to either improve or aggravate pathological consequences (13, 34–41). In models of SE and acquired epilepsy, suppression of CSF1R signaling was associated with decreases in SE-induced neuronal loss and seizure frequency (13, 34) but also with an exacerbated acute seizure response to kainate (40, 41) and increased seizure frequency in epileptic mice (40). In a mouse model of Theiler's murine encephalomyelitis virus-induced epilepsy microglia depletion intensified the seizure severity and resulted in fatal encephalitis (39). This body of evidence along with the finding the lack of microglia due to homozygous mutations in the CSF1R gene are associated with brain malformations, developmental delay, and epilepsy in humans (17), and new findings that microglia directly control neuronal activity through a negative feedback mechanism (41), suggest that microglia may be important to prevent and/or control the generation of epileptic networks. We speculate that the long-lasting suppression of microgliosis after SE in our study may have allowed or perhaps exacerbated epileptogenesis and worsen memory dysfunction, though it is also possible that PLX treatment may slow or prevent epileptogenic processes. Therefore, a limitation of our study is that we did not determine the impact of PLX3397 or a combination treatment of PLX3397 with an anti-seizure drug during the period of epileptogenesis using electroencephalographic (EEG) recordings. These would aid in differentiating the potential role the experimental treatments (PLX3397 and SC) have on the extent of hippocampal microgliosis, spontaneous seizure duration and frequency, and the cognitive defects within the same animals. In addition, EEG recordings could help determine the relation between seizure development and cognitive decline, as seizures can have profound consequences on the behaviors evaluated in the NOR and BM test. Our future studies will investigate these aspects.

Consistent with previous studies (13, 22), we found that the PLX3397 diet promoted a decrease of ~60% in the population of microglial cells both in control and SE-treated rats. We focused on the CA1 hippocampus as a representative brain area because we found the most robust SE-induced increases in microgliosis in this region (6–9) and because these microglial changes closely paralleled memory loss (5–7). In a mouse model of AD, treatment with a CSF1R inhibitor reduced microgliosis by 80% in the hippocampus, cortex, and thalamus (19), while in healthy aging mice, this effect was close to 99% (16, 22, 42). Other studies also support that the extent of microglial suppression with the CSF1R inhibitor is consistent when given under physiological conditions but highly variable in neurological disorders including AD, Parkinson's disease, ischemia, and epilepsy (13, 19, 25, 43) which suggest that the activation of CSF1R signaling or the levels of CSF1 may be differently altered under variable pathological conditions, including during the period of epileptogenesis. A limitation of our study is that we were not able to measure the extent of potential alterations in CSF1R expression or its phosphorylation/activation status during epileptogenesis due to the lack of antibodies with appropriate specificity for the rat tissues. Spatial and temporal information about the status of CSF1R signaling would be necessary to determine the extent of the contribution of this pathway to SE-induced microgliosis and the neuropathology and pathophysiology of epilepsy. This information would help guide the selection of a relevant time window along with an appropriate dose-response test to assess different levels of microglial suppression with pharmacological tools such as PLX3397 or its analogs during epileptogenesis and in chronic epilepsy.

Thus, it is not known how exactly SE impacts the activation of CSF1R signaling in microglia and which specific functions are altered at the time point evaluated in this study. The decrease in microgliosis indicates that PLX3397 suppressed the survival and proliferation of these cells. However, we also found a shift in the morphology of the remaining microglia. In both control and SE groups, treated with PLX3397 an increase in the abundance of hypertrophic microglia suggests that the function of these remaining cells may be changed. This is further supported by the lack of changes in the hippocampal protein levels of CX3CR1 between PLX3397- and regular chow-treated groups despite a reduction of 60% in the total numbers of microglia in the hippocampus, which suggest a potential compensatory increase in the expression of this receptor in the remaining microglia. While it is not definitively known how different microglial shapes associate with specific functions some studies support that bushy/amoeboid microglia may be highly phagocytic due to the presence of high levels of phagocytic lysosomal markers (44–47) and that hypertrophic microglia may be inflammatory (48, 49).

Interestingly, we found that PLX3397 also reduced the SE-induced increases in the hippocampal protein levels of GFAP suggesting a decrease in astrogliosis (Figure 7). This finding further supports that these two cell types communicate which each other under pathological conditions (50). Microglia and astrocytes communicate through a number of molecules including complement proteins (32, 50), which are upregulated in epilepsy (9). Reactive microglia produce and release the complement protein C1q which in turn triggers astrocytes to release C3a which then binds to its receptor (C3aR) in astrocytes, thereby producing a regulatory loop (32, 50). Although PLX3397 reduced astrogliosis, the levels of C3 protein in whole hippocampal homogenates remained unchanged. This suggests the possibility that other pathways or cell types may be regulating C3 levels in response to SE, or that the immunoblot approach used in our study missed changes that may be regional within the hippocampal formation, as recently shown by Wei et al. (32). Furthermore, due to the tight association between SE-induced increases in C3 complement activation and memory decline following pilocarpine-induced SE in rats (5, 6) we speculate that the high levels of C3 still present in both SE groups (PLX3397 and standard chow) may be modulating the memory loss.

In sum, our study shows that dietary treatment with the CSF1R inhibitor PLX3397 during the period of SE-induced epileptogenesis in the rat does not alter memory functions even though microglial numbers were significantly reduced. Our findings do not support or reject pro- or anti-epileptogenic roles for microglia during SE-induced epileptogenesis, only that reducing microgliosis by 60% in the hippocampus was not sufficient to attenuate SE-induced memory defects. Therefore, the answer to whether microgliosis itself underlies cognitive decline during epileptogenesis is still in need of further investigation. Future studies will focus on a comprehensive characterization of CSF1R receptor expression and activation status needed to understand its contribution to microglial proliferation and function in epilepsy, and to determine whether a full depletion of microglia is necessary to attenuate or prevent epileptogenesis.

## Data Availability Statement

The raw data supporting the conclusions of this article will be made available by the authors, without undue reservation.

## Ethics Statement

The animal study was reviewed and approved by Purdue Institutional Animal Care and Use Committee (Protocol #1309000927).

## Author Contributions

SW-J and AB contributed to idea development and experimental design of the study. SW-J conducted experiments, analyzed data, made figures, and wrote the first draft of the manuscript. AS performed behavioral and biochemical experiments and analyzed behavioral data. KS performed biochemical experiments and analyzed behavioral data. AB wrote sections of the manuscript. All authors read and approved the submitted version.

## Conflict of Interest

The authors declare that the research was conducted in the absence of any commercial or financial relationships that could be construed as a potential conflict of interest.

## Supplementary Material

Supplemental Figure 1 PLX3397 in chow (PLX; 50 mg/kg per day) did not suppress microgliosis in five rats that sustained the same level of pilocarpine-induced status epilepticus (SE) and had similar body weights. **(A)** Behavioral seizures were monitored for 100 minutes after SE induction and scored according to the Racine scale (1: rigid posture, mouth moving; 2: tail clonus; 3: partial body clonus, head bobbing; 4: rearing; 4.5: severe whole body clonic seizures while retaining posture; 5: rearing and falling; 6: tonic-clonic seizure with jumping or loss of posture). Three SE groups are shown: SE +standard chow (SC) (SE+SC), SE+PLX, and SE+PLX Non-responder (Non or N). **(B)** Time to first seizure (level 3). **(C)** Time to SE (level 6). **(D)** Graph shows the daily body weight of rats (days 0-21) from all SE groups. **(E)** Quantification of IBA1 positive cells per mm^2^ in CA1 sr. Data analyzed by Kruskal-Willis test with Dunn's multiple comparisons **(A)**, one-way ANOVA with Dunnett's multiple comparisons **(B,C,E)**, and two-way ANOVA with Dunnett's multiple comparisons **(D)**. Data are shown as mean ± SEM. SE+SC, *n* = 14; SE+PLX, *n* = 9; SE+PLX Non-Responder, *n* = 5. ^*^*p* < 0.05.
