# Supplementary figures and images for: Suppression of Microgliosis With the Colony-Stimulating Factor 1 Receptor Inhibitor PLX3397 Does Not Attenuate Memory Defects During Epileptogenesis in the Rat
Source: Front Neurol. 2021 Jun 3;12:651096. doi: 10.3389/fneur.2021.651096 (PMC8209304; doi:10.3389/fneur.2021.651096)

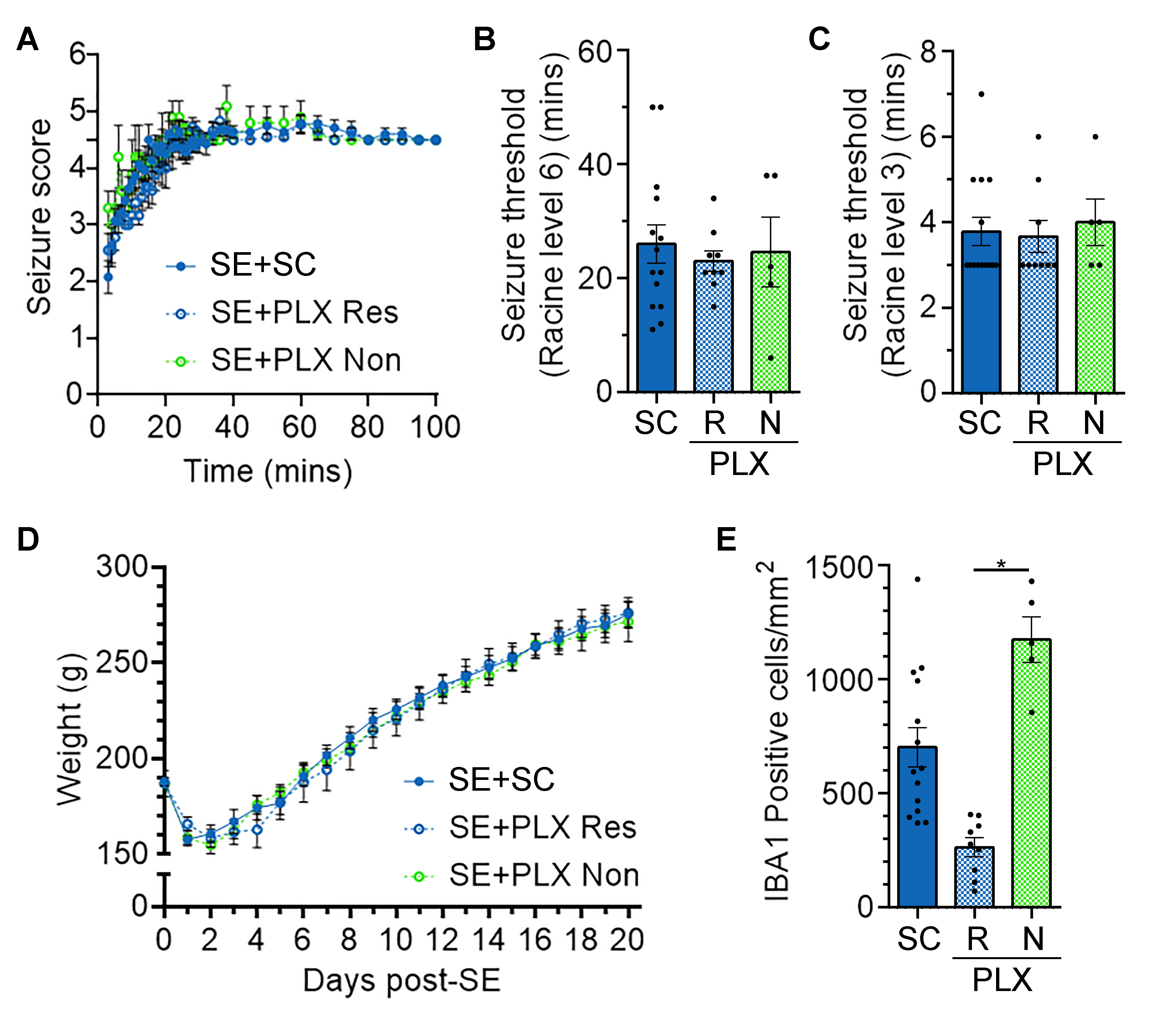

Supplement: Supplemental Figure 1 — PLX3397 in chow (PLX; 50 mg/kg per day) did not suppress microgliosis in five rats that sustained the same level of pilocarpine-induced status epilepticus (SE) and had similar body weights. (A) Behavioral seizures were monitored for 100 minutes after SE induction and scored according to the Racine scale (1: rigid posture, mouth moving; 2: tail clonus; 3: partial body clonus, head bobbing; 4: rearing; 4.5: severe whole body clonic seizures while retaining posture; 5: rearing and falling; 6: tonic-clonic seizure with jumping or loss of posture). Three SE groups are shown: SE +standard chow (SC) (SE+SC), SE+PLX, and SE+PLX Non-responder (Non or N). (B) Time to first seizure (level 3). (C) Time to SE (level 6). (D) Graph shows the daily body weight of rats (days 0-21) from all SE groups. (E) Quantification of IBA1 positive cells per mm2 in CA1 sr. Data analyzed by Kruskal-Willis test with Dunn's multiple comparisons (A), one-way ANOVA with Dunnett's multiple comparisons (B,C,E), and two-way ANOVA with Dunnett's multiple comparisons (D). Data are shown as mean ± SEM. SE+SC, n = 14; SE+PLX, n = 9; SE+PLX Non-Responder, n = 5. *p < 0.05. [file Image_1.JPEG]
